# Prospective multicentre multifaceted before-after implementation study of ICU delirium guidelines: a process evaluation

**DOI:** 10.1136/bmjoq-2019-000871

**Published:** 2020-09-18

**Authors:** Zoran Trogrlic, Mathieu van der Jagt, Theo van Achterberg, Huibert Ponssen, Jeannette Schoonderbeek, Frodo Schreiner, Serge Verbrugge, Annemieke Dijkstra, Jan Bakker, Erwin Ista

**Affiliations:** 1 Department of Intensive Care, Erasmus Medical Center Rotterdam, Rotterdam, The Netherlands; 2 Public Health and Primary Care, Academic Centre for Nursing and Midwifery, Katholieke Universiteit Leuven, Leuven, Belgium; 3 Department of Intensive Care, Albert Schweitzer Ziekenhuis, Dordrecht, The Netherlands; 4 Department of Intensive Care, Ikazia Ziekenhuis, Rotterdam, The Netherlands; 5 Department of Intensive Care, IJsselland Ziekenhuis, Capelle aan den IJssel, The Netherlands; 6 Department of Intensive Care, Sint Franciscus Gasthuis & Vlietland, Rotterdam, The Netherlands; 7 Department of Intensive Care, Maasstad Ziekenhuis, Rotterdam, The Netherlands; 8 Department of Pulmonology and Critical Care, New York University - Langone, New York, New York, USA; 9 Department of Pulmonology and Critical Care, Columbia University Medical Center, New York Presbyterian, New York, New York, USA; 10 Department of Intensive Care, Pontificia Universidad Catolica de Chile, Santiago, Chile; 11 Department of Pediatric Surgery, Intensive Care Unit, Erasmus MC Sophia Kinderziekenhuis, Rotterdam, The Netherlands; 12 Department of Internal Medicine, Nursing Science, Erasmus MC, Rotterdam, The Netherlands

**Keywords:** critical care, implementation science, clinical practice guidelines, evaluation methodology, evidence-based practice

## Abstract

**Objective:**

We aimed to explore: the exposure of healthcare workers to a delirium guidelines implementation programme; effects on guideline adherence at intensive care unit (ICU) level; impact on knowledge and barriers, and experiences with the implementation.

**Design:**

A mixed-methods process evaluation of a prospective multicentre implementation study.

**Setting:**

Six ICUs.

**Participants:**

4449 adult ICU patients and 500 ICU professionals approximately.

**Intervention:**

A tailored implementation programme.

**Main outcome measure:**

Adherence to delirium guidelines recommendations at ICU level before, during and after implementation; knowledge and perceived barriers; and experiences with the implementation.

**Results:**

Five of six ICUs were exposed to all implementation strategies as planned. More than 85% followed the required e-learnings; 92% of the nurses attended the clinical classroom lessons; five ICUs used all available implementation strategies and perceived to have implemented all guideline recommendations (>90%). Adherence to predefined performance indicators (PIs) at ICU level was only above the preset target (>85%) for delirium screening. For all other PIs, the inter-ICU variability was between 34% and 72%. The implementation of delirium guidelines was feasible and successful in resolving the majority of barriers found before the implementation. The improvement was well sustained 6 months after full guideline implementation. Knowledge about delirium was improved (from 61% to 65%). The implementation programme was experienced as very successful.

**Conclusions:**

Multifaceted implementation can improve and sustain adherence to delirium guidelines, is feasible and can largely be performed as planned. However, variability in delirium guideline adherence at individual ICUs remains a challenge, indicating the need for more tailoring at centre level.

## Introduction

Delirium is strongly associated with intensive care unit (ICU) length of stay, mortality and long-term cognitive and functional impairments.^1–4^ Previous studies have indicated that delirium can be reduced by using less sedation and avoiding use of benzodiazepines, early weaning from mechanical ventilation, and early physical therapy and mobilisation.^3 5 6^ Those evidence-based interventions are summarised in the 2013 Pain, Agitation and Delirium (PAD) guidelines^7^ and more recently in the updated Pain, Agitation/Sedation, Delirium, Immobility and Sleep Disruption guidelines of the Society of Critical Care Medicine.^8^ Implementation of PAD guidelines in the ICU setting was mostly done in previous studies with high levels of resources, and with dedicated research personnel using the ‘Awakening and Breathing Coordination, Choice of drugs, Delirium monitoring and management, Early mobility, and Family engagement’ bundle.^9–13^

Recently, we published the results of a multicentre implementation study aimed to implement delirium-oriented recommendations derived from the Dutch ICU Delirium Guidelines^14^ and the 2013 PAD guidelines.^7^ In this study named the ‘ICU DElirium in Clinical PracTice Implementation Evaluation’ (iDECePTIvE) Study, a multifaceted implementation programme based on preimplementation assessment of barriers was developed and evaluated.^15–17^ The overall results showed an improved adherence to delirium guidelines and recommendations which have resulted in decreased levels of brain dysfunction, meaning reduced delirium duration and a lower number of coma days.^17^ However, variable guideline adoption among different sites is a well-known phenomenon,^18^ which may also provide insights on factors that enhance effective implementation and guideline adoption. Therefore, this process evaluation study aimed to further zoom in into the implementation interventions to get insight into the determinants and indicators of success or failure of the implementation programme and to provide more detailed background information on the entire implementation process.

We explore the following four issues: (1) Actual exposure to the implementation programme at the individual ICU level; (2) Effects of the implementation programme on guideline adherence at the individual ICU level and it’s sustainability after 6 months; (3) Impact of the implementation programme on implementation barriers and knowledge among ICU professionals over time; and finally, (4) The experiences of the site-specific implementation teams with the implementation programme.

## Methods

### Design, setting and participants

This was a mixed-methods process evaluation of a multicentre prospective prepost implementation study (iDECePTIvE). This report adhered to the Standards for Reporting Implementation Studies (StaRI) statement.^19^ See online supplemental file 1 for the completed StaRI checklist. The Implementation Model of Change of Grol and Wensing was used to structure the guideline implementation.^20^ The details of the study design and methods have been reported previously.^15 17^ Briefly, data for performance indicators (PIs) on adherence to guideline recommendations from the PAD guidelines related to delirium were collected in four phases, defined as follows: first phase (T1, baseline period); before implementation, usual care was evaluated, second phase (T2); after implementation of delirium screening tools, third phase (T3): after implementation of delirium treatment and prevention measures, and fourth phase (T4); 6 months after the implementation to assess the sustainability. Whereas the findings of the iDECePTIvE Study were based on the comprehensive data of all ICUs combined,^17^ this process evaluation is a subanalysis of data and expands on the findings at the individual site (ICU) level and the addition of results on short-term sustainability of guideline adoption.

10.1136/bmjoq-2019-000871.supp1 Supplementary data

The major implementation strategies of the implementation programme were education, audit and feedback, and reminders, as previously described.^17^ In brief, education was provided in the form of web-based e-learning (http://delier.intensivecare.me/index.html). Education was provided first in phase II during implementation of screening for delirium and thereafter in phase III, where it focused on the contents of delirium prevention and management guidelines. The content of e-learning was classified according to general information about ICU delirium, delirium screening, prevention and integral management of delirium, where family participation was a prominent and important issue. The text and video about patient experiences were also incorporated in e-learning and information about global delirium research was shared on the e-learning website. In addition to e-learning, didactic classroom educational sessions for nurses were held, aimed to educate and discuss the questions raised about delirium screening and protocols, and to provide more information about the implementation and practical application of the protocols. Presence in the classroom education sessions was recorded and the lessons were continued until more than 75% of the nurses attended this education. The physicians were not required to be present at the clinical classroom lessons. To maintain process changes and implementation effects, all new employees were educated with e-learning. During phase II of the study educational spot checks of delirium screening (target was four spot check moments per nurses vs local experts) were performed. Audit and feedback were applied in two ways during phase II and III: (1) Using posters with delirium screening adherence and prevalence of delirium of the individual ICU (phase II), which were presented to the ICU staff of the separate ICU every quarter;^15^ and (2) Using a so-called Implementation Readiness Test (IRT, phase III; explained in the next paragraph). During phase II, posters on delirium screening were presented to the ICU staff of the separate ICUs every quarter. These posters presented the actual adherence rates of the individual ICU and the mean of all centres to delirium screening for comparison and visualised the predefined adherence-level aim of 85%.^15^ To further facilitate the use of the guidelines in daily practice and to sustain the implementation, an ICU Delirium app was developed as an implementation facilitator (link: http://icudelierapp.nl). The app was focused on the healthcare professional who received advice on additional management regarding delirium in a certain patient using a stepwise evaluation of the current status of the patient and current management. The app was released in January 2015. Reminders were used as the standard notifications and flow charts for delirium screening and management in the electronic patient files system. An information leaflet and a poster for family members of ICU patients were used to inform them about the identification, prevention and treatment of delirium in an attempt to further enhance and stimulate structural attention for delirium by next of kin and stimulate discussions with care providers.

### Data collection

#### Actual exposure to the implementation programme

To be able to follow the implementation progress at different sites and to provide the sites with implementation feedback, we drafted an implementation process check tool, which we named the ‘Implementation Readiness Test’. The IRT was applied three times in 8 months during the audit visits in phase III to evaluate the current status and progress of implementation as perceived by the local implementation team. The IRT consisted of two parts: (1) Assessment of application of the number of implementation strategies by the local study team; and (2) The local study team’s perception of the extent to which the guideline recommendations were actually implemented into clinical practice. Based on the IRT, a feedback and action plan at site level including the priorities for each site, was made. Follow-up IRTs were done twice approximately every 3 months. The study team also used IRTs to monitor the progress of implementation at all sites, and was used to monitor and semiquantitatively assess implementation progress.

#### Effect of the implementation programme on guideline adherence at ICU level

All consecutive adult ICU patients were included. Adherence rates to the guideline recommendations at site level were assessed with seven PIs.^17^ In addition to the previous paper,^17^ we now added the data on the sustainability of the adherence changes 6 months after implementation phase III.

#### Impact of the implementation programme on knowledge and implementation barriers

Beliefs, attitudes, practices, knowledge, guideline implementation barriers, and facilitators for nurses and physicians of the ICUs were assessed twice, both before T1, and after the guideline implementation (T3). Details of the questionnaire were previously published.^16^

#### Experiences with the implementation programme

In order to explore the experiences of local implementation teams, we organised interviews at each site after completion of phase III. The interviews were semistructured with predefined questions about the experiences with the implementation programme and its components (online supplemental file 2). We also asked the members of local implementation teams to provide the study implementation management team with feedback and to give their opinions on the success of implementation, barriers perceived during execution of the implementation programme and the satisfaction with the programme. All interviews were audio-recorded and conducted by the same moderator.

### Data analysis

#### Quantitative data

Data regarding the actual exposure to the individual elements of the implementation strategies were presented as percentages or absolute numbers. The questionnaires were distributed before phase I and after implementation. For the questions about ‘attitude and perceptions’ and the ‘current practices’ we used the questions with dichotomous answer options yes/no or agree/disagree (from the 5-point Likert Scale statements where options: 1=strongly disagree; 2=disagree; 3=neutral; were marked as disagree and options 4=agree; 5=strongly agree were marked as agree). Barriers for these dichotomous questions were considered to be present if <50% of the respondents gave an answer implicating support for the issue pertaining to that statement. Barriers for delirium guideline and guidelines in general adherence were assessed with 6-point Likert Scales (no agreement=0 and maximum agreement=5). Mean scores of ≥3 were considered to indicate agreement with statements and was considered as a barrier.^16^ A delirium knowledge score was calculated per respondent, defined as the percentage of correct answers. A mean delirium knowledge score below 70% was considered as a barrier regarding knowledge at the group level (eg, ICU, nurses, physicians). Student’s t-test (for two groups) and one-way analysis of variance (for three groups) was used to test the differences per ICU before versus after implementation. Weighted frequencies and proportions of the total ICU patient days contributed by each ICU were used to describe the adherence to the seven PIs and were described at ICU level and stratified by the four periods. The relative change in adherence difference between the baseline (T1) and the follow-up (T4) for each ICU and each guideline recommendation was given as ΔT4-T1 and the crude adherence numbers for T1 and T4 were reported.

#### Qualitative data

Associations between guideline adherence and exposure to implementation strategies were explored qualitatively by visual inspection. The interviews were transcribed verbatim and summaries of the interviews were sent to the participants to check for accuracy and validity of transcriptions. The moderator of the interviews had also analysed the data through reading and rereading interviews in order to obtain the essence of the whole. A thematic content analysis approach was used in searching themes.^21^ Next, themes were labelled, coded and defined as: factors of implementation success, experience in collaboration with the study implementation team and lessons learnt for future implementations. Reliability checks were done by a second researcher, and discussed and resolved in case of any unclarities.

## Results

All available staff working at the ICUs, 81 physicians (range within ICUs: 5 to 31) and 409 nurses (range: 35 to 125 per ICU), was targeted to participate in the implementation programme. Depending on the number of ICU beds, the local implementation expert teams consisted of 2–11 ICU professionals. All ICUs were visited by the study management team at least seven times. One site (ICU 4) was visited 10 times due to challenges in the implementation caused by changes in RNs involved.

### Actual exposure to the implementation programme

The average self-recorded time spent on both e-learnings was about 45 min per person per e-learning. Classical clinical lessons for delirium screening and PAD recommendations were repeated several times (about 45 min for each lesson). The majority of nurses (n=375; 92%) attended the clinical classroom lessons. During study phase II educational spot checks of delirium screening (nurses vs local experts) were performed as intended (four spot check moments per nurse).

Table 1 shows an overview of three completed IRT forms (filled in approximately 3 months apart), just before the T3 data collection period. Total score just before the start of T3 data collection was for both parts of IRT between 90% and 100% and had overall improved compared with the first assessment 6 months earlier. Five ICUs used all implementation strategies and implemented all guideline recommendations. Only ICU 4 lagged behind and used 81% of the available implementation strategies and implemented only 67% of the advised protocol recommendations in daily practice.

**Table 1:** Implementation Readiness Test (exposure in number of intensive care units (ICUs))

Implementation strategy	Norm/requirements	IRT 1*	IRT 2	IRT 3
**Part 1: Execution of implementation strategies**				
Education: Learning Part 1 screening	≥75% of nurses have completed the e-learning?	6†	6	6
Education: e-learning Part 1 screening	≥75% of physicians have completed the e-learning?	4	5	6
Education: e-learning Part 2 - treatment and preventive protocol	≥75% of nurses have completed the e-learning?	2	2	6
Education: e-learning Part 2 - treatment and preventive protocol	≥75% of physicians have completed the e-learning?	2	3	6
Clinical lessons screening	New employees are trained around delirium management?	3	4	4‡
Educational outreach				
Spot checks screening	There are at least four spot checks done by a nurse?	5	5	5
Quality control screening	This is scored by the experts? (interobserver variation)?	3	4	5
Local implementation teams				
	Local implementation team is multidisciplinary (at least: intensivist, IC nurse and possibly: psychiatrist/neurologist/geriatrician/physical therapist)?	6	6	6
	There were at least two consultations between local implementation team members (since beginning of the study) and there are agreements on implementation?	4	5	6
	It was agreed (preferably also recorded) who is responsible for which part of the implementation.	6	6	6
Local opinion leaders	It is clear who the implementation team members are and who is a contact for delirium in general and the study in particular?	5	5	6
Audit and feedback				
Indicators poster screening and incidence	1. Are the posters visible?	5	6	6
	2. Are those discussed in the management team?	2	5	6
Decision support				
Laminated pocket cards screening CAM-ICU or ICDSC	Are pocket cards present for nurses and physicians?	5	6	6
	Pocket cards are used in practice?	3	4	5§
Reminders	There are reminders regarding screening and management of delirium (if available, pop-ups PDMS for screening)	6	5	6
Focus groups/barrier analysis	Bottlenecks are discussed in local multidisciplinary meetings at the ICU level and is the implementation aimed to address them?	2	3	5
**TOTAL (of max 99**)		**69** (**70%**)	**80** (**81%**)	**96** (**97%**)
**Part 2: Implementation of protocol**				
PDMS (patient demographic management system)	Is PDMS modified and helpful for delirium screening?	5	5	5¶
Treatment delirium	Are the 4HS 4TS used in practice regularly if delirium screening result is a positive one (new delirium)?	0	3	5
	Is it clear what the drug treatment for delirium (according to protocol) is?	4	6	5
	Is medication sometimes modified following the screening?	5	6	6
	Are the non-pharmacological measures optimised before starting medication?	2	3	5
Prevention of delirium: physical therapy and early mobilisation	Physical therapy: there are structural arrangements with physical therapist and there is agreement about how to provide early physical therapy and mobilisation?	2	3	6
	Mobilisation of patients is basically addressed by daily patient rounds and this is implemented in the daily rounds?	4	5	6
	Is department policy that seeks to mobilise ventilated patients if possible?	3	4	5
Prevention: sleep hygiene	Is there a protocol regarding sleep promotion?	3	6	6
	Used this protocol and regularly followed in practice?	0	5	5
	Sleep protocol contains at least the next recommendations: lights off or muted overnight, strive for sleep (no standard rounds running if not necessary), and use of earplugs?	5	5	6
Prevention: psycho hygiene (among other, reducing sensory deprivation)	Is there a structural focus on using eyeglasses and of hearing aid if applicable throughout the ICU admission?	4	5	6
Evaluation of pain-sedation-delirium	Daily delirium screening is implemented and ‘going well‘?	3	4	6
	The coordination of delirium, sedation and pain management is implemented in any way in the daily rounds (eg, visit form)?	4	5	6
	Daily rounds checklist is implemented and used?	3	4	5
Sedation	Sedation with midazolam (or other benzodiazepines) by continuous infusion is avoided, and alternative sedation (analgo-sedation with opiate and possibly clonidine/dexmedetomidine/propofol targeting addressable patient comfortable?) is used?	4	5	6
Family engagement	Is there a leaflet about delirium for family?	4	4	6
	Family of the ICU patient is getting the opportunity to contribute in identifying and/or treatment of delirium (eg, to help with washing, etc)?	3	5	6
	Poster about family engagement by delirium is presented in the family room?	1	2	5
**TOTAL (of max 113**)		**59** (**52%**)	**84** (**74%**)	**106** (**94%**)

*IRT, Implementation Readiness Test, drafted to measure the actual exposure to implementation strategies as perceived by the local study team. All three IRT overviews were made in phase III during the implementation of guideline (total time=10 months). The last one IRT overview was made just before the start of third data collection period (T3).

†The numbers indicate the number of sites that have implemented the item in daily practice.

‡Not applicable for two ICUs because there were no new employees during previous period.

§Not applicable for one ICU because the information as given in pocket cards was integrated in PDMS.

¶Not applicable for one ICU because no PDMS system was available.

### Effect of the implementation programme on guideline adherence at the level of the participating ICUs and sustainability

The fourth data collection period served to assess sustainability of the implementation, and included an additional 519 patients (2727 days) (online supplemental file 3) next to the 3930 patients from the previous three phases. Only the percentage of mechanically ventilated patients was higher (51%) than in the preceding three phases (respectively, 42%; 39%; 50%) as previously published.^17^

**Figure 1:**
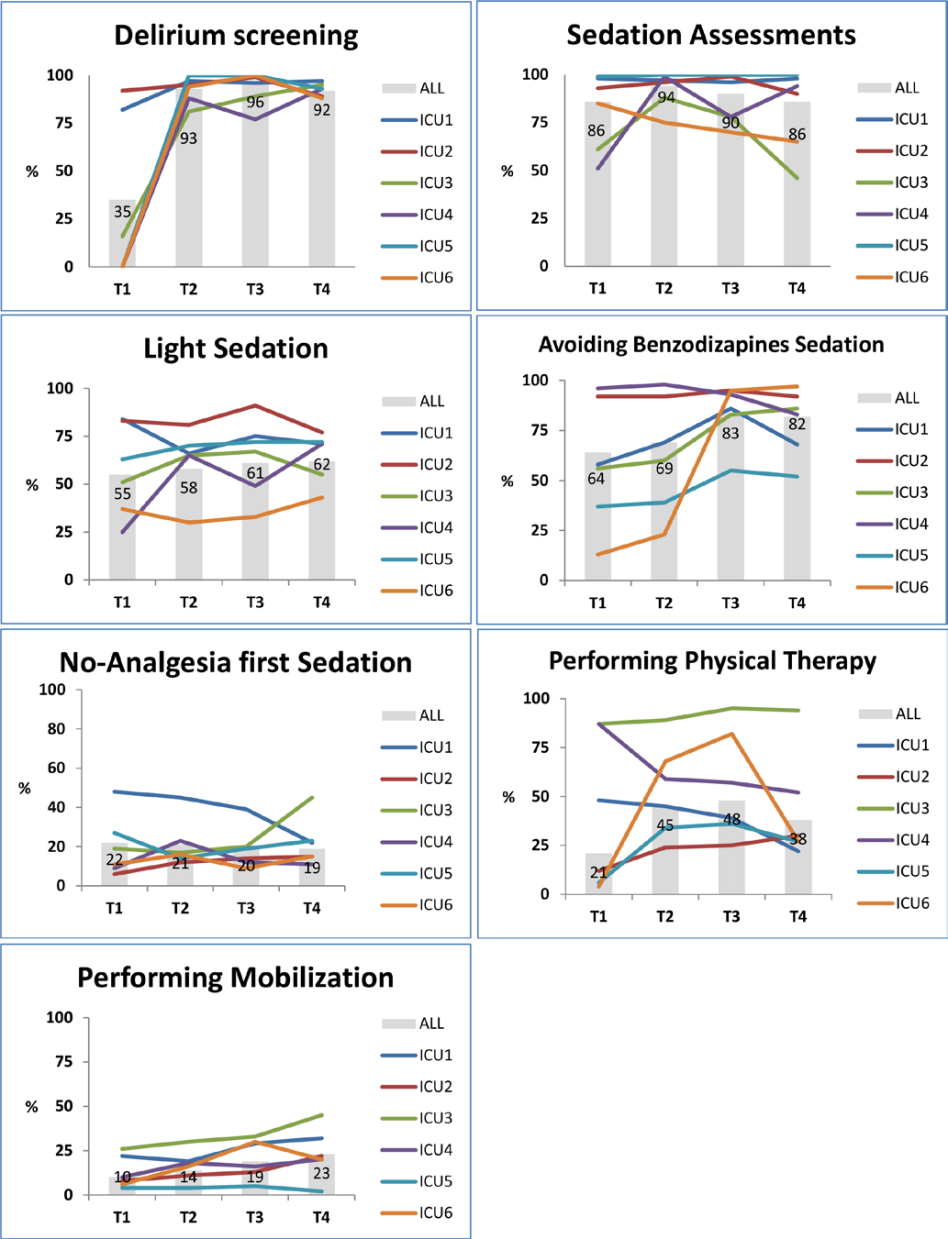
Adherence to process indicators over the study periods.

Figure 1 displays the changes on adherence to the PIs in the different ICUs over time. Weighted percentages of the total ICU patient days contributed by each ICU of all PIs for all four measurement periods are given in online supplemental file 4. Adherence to the seven PIs improved overall and this improvement was sustained 6 months after active implementation support by the study management team had been terminated. Four PIs improved by more than 10%. The adherence to delirium screening (ΔT4-T1) improved most significantly with +57%, followed by avoiding benzodiazepine sedation (+18%); performing Physical Therapy (PT) (+17%); and performing mobilisation (+13%). Sedation assessments were improved during implementation, but the improvement of +8% was not sustained after implementation and dropped to the initial adherence level of 86%. Performing physical therapy initially improved by 27%, but dropped to 17% in T4. Light sedation improved slightly by 7%.

Despite the overall improvement on process indicators, not all ICUs succeeded in adherence improvement for all PIs. In contrast and remarkably, decreases in adherence of more than 10% were measured on four PIs between baseline and follow-up. These were: sedation assessments (ICU 3 = −15%; and ICU 6 = −20%); light sedation (ICU 1 = −13%); avoiding benzodiazepine sedation (ICU 4 = −13%); and performing physical therapy (ICU 1 = −26%; and ICU 4 = −35%).

There was no clear relationship between centre-specific adherence changes and clinical outcomes changes per ICU, similar to the overall results. Online supplemental file 5 shows the changes of clinical outcomes per ICU per study phase.

### Impact of the implementation programme on knowledge and implementation barriers

In total, 360 (69%) and 264 (50%) healthcare professionals completed the survey at T1 and T3, respectively. There were no differences between the participants at T1 and T3 in years of experience, work assignment and age (see online supplemental file 6). Delirium knowledge test scores improved modestly at a group level from 62.9 (SD=13.3) before to 65.1 (SD=13.1) after the implementation. However, these differences were only present in three of the ICUs (ICU 1: from 65% to 67%; ICU 2: from 62% to 64%; and ICU 6: from 60% to 66%) that succeeded in obtaining improved knowledge scores, while we found no differences in exposure to education for these three ICUs.

From all barriers identified through the survey before the implementation a quarter was not resolved by the implementation programme. The perception that ‘delirium is not preventable’ was not resolved. This may have affected, for example, the use of earplugs for the night. Also, the perception that ‘routinely addressing delirium in daily rounds can still be improved after the implementation’ was not resolved, and finally, the satisfaction of nurses about delirium treatment did not improve (table 2).

**Table 2:** Comparison of barriers found by the first survey versus the results of the second survey

	Before	After
(A) Attitudes and perceptions	%*	
Delirium occurrence and importance		
Delirium is preventable	21	15
Screening	%*	
Is a nurse capable to identify delirium with a validated delirium screening instrument?	34	80
Collaboration	%*	
When I as nurse suspect a patient to be delirious, I am satisfied with delirium treatment	47	40
When I as physician suspect a patient to be delirious, the nurse is satisfied with delirium treatment	42	11
Collaboration between doctors and nurses with regard to delirium at the ICU can be improved by better screening	65	30
Collaboration between doctors and nurses with regard to delirium at the ICU can be improved by routinely addressing delirium in daily rounds	74	78
(B) Current practices		
Delirium screening	%*	
In the ICU unit where I work the following delirium screening scale is in use:		
CAM-ICU (Before: n=210; in only two hospitals / After: n=119)	58	45
ICDSC (before: n=3/after: n=104)	<1	39
Delirium prevention		
Earplugs for the night	8	24
Family visits as much as possible	50	61
(C) Guideline adherence (n=136)		
If I follow the guideline recommendations, it is likely that my patients would not receive optimal care†	3.1 (1.0)	1.9 (1.1)
I do not wish to change my delirium care practices, regardless of what delirium guideline recommends†	3.7 (1.0)	1.4 (1.0)
I don’t have time to use this guideline†	3.5 (0.9)	1.7 (1.0)
This guideline is cumbersome and inconvenient†	3.0 (1.1)	2.0 (1.1)
(D) Guideline adherence in general (n=128)		
Generally, guidelines are cumbersome and inconvenient†	3.0 (0.9)	2.2 (0.9)
Guidelines are difficult to apply and adopt to my specific practice†	3.1 (0.9)	2.0 (0.9)
Guidelines interfere with my professional autonomy†	3.3 (0.9)	1.7 (0.9)
Generally, I would prefer to continue my routines and habits rather than to change† based on practice guidelines†	3.3 (1.0)	1.9 (0.9)
I am not really expected to use guidelines in my practice setting†	3.7 (0.9)	1.4 (1.0)

*= % agreement (= %YES answers or % of the sum of agree and strongly agree answers (from the 5-point Likert Scale statements)). Barriers depends on the question formulation. For positive formulated the barrier is ≤50% and negative formulated the barrier is ≥50%.

†= mean and SD based on the six-point Likert Scale. Mean score of ≥3 was considered to indicate agreement with statement=Barrier.

CAM-ICU, Confusion Assessment Method for the ICU; ICDSC, Intensive Care Delirium Screening Checklist; ICU, intensive care unit.

### Experiences with the implementation programme

Overall, the members of the local implementation teams found the implementation programme as very successful. The most important themes were the encouragement of the local implementation team by the implementation management team, change of culture with regard to the attitude of professionals towards delirium as a form of brain failure and the improvement in collaboration with other (not ICU) disciplines due to the implementation. Despite the belief that a positive change in practice around delirium management had been made, the application of delirium preventive interventions still deserved more attention. A more detailed report of the semistructured interview findings about experiences with the implementation programme is given in online supplemental file 7.

## Discussion

In this process evaluation of a multicentre delirium guidelines implementation programme, we found that all ICUs, except for one, were exposed to more than 90% of the implementation strategies. The implementation of the delirium guideline using the tailored implementation programme was feasible and successful in resolving the majority of barriers found before the implementation. It resulted in improved knowledge about delirium, and it improved the daily process of care at six ICU sites as defined by seven PIs, which generally proved sustainable when measured after 6 months. However, the results on the PIs showed a considerable variation in guideline adoption across the six ICUs. Experiences with the implementation support from the research coordinators were favourable, but continued support and coaching was deemed necessary to support the implementation interventions throughout the study.

Despite the general improvements in process of care outcomes, our data do not allow for conclusions regarding an association of individual implementation strategies and adherence changes because all sites largely executed the implementation as intended. Different entry levels of adherence and variation in time also make it difficult to compare the changes in time. However, the wide variation in guideline adoption may be an argument that there is still room for more centre-level tailoring. This paper should be regarded as a companion article to our previous paper that included clinical outcomes,^17^ but with more in-depth focus into the processes of the implementation, for readers to be able to construct their own implementation based on local possibilities, in an ICU setting.

We have identified relevant differences in the ‘dose’ of implementation for individual PIs. Only for delirium screening the norm (goal ≥85%) was set before the implementation and repeated feedback about performance on this PI was given during the implementation phase. In daily practice there was more focus and education on this topic (separate e-learning and classical lessons, and spot checks), and there were specific patient data management system adjustments and delirium screening quality checks. Setting a clear adherence-level goal in combination with using audit and feedback for all PIs may have resulted in an increased level of adherence. Positive effects of audit and feedback on professionals’ intentions to improve practice have been empirically evaluated.^22^ In our study the feedback data were collected and given only for delirium assessment and incidence of delirium. We suggest this was a facilitator in improving adherence in combination with electronic reminders to create continued awareness for delirium assessment and presence of delirium.

Even though all sites were exposed to the same implementation programme there were differences in the adherence changes across the sites. One of the possible explanations in the variability in adherence to the implementation programme is the fact that there were other implementation projects, and organisational changes going on at the different sites which diverted the attention of the physicians, nurses and managers. During the study, two ICUs underwent organisational changes such as opening a medium care unit at the ICU, and separating medium care and ICU care patients at different units (ICU 1 and ICU 6). Such changes could be the reason behind the increased number of mechanically ventilated patients over the four study periods (baseline 42%–51% in follow-up). But more importantly, we did not assess culture, organisational aspects and other context-related factors before implementation across multiple sites which may have shed light on the variable adoption. Retrospectively, the Consolidated Framework for Implementation Research (CFIR)^23^ could have been a helpful implementation model: in contrast to the implementation model of change of Grol and Wensing,^20^ the CFIR model operationalised the organisational context by two dedicated domains: ‘inner setting’ (local culture, leadership engagement, implementation climate, etc), and ‘outer setting’ (patients’ needs and resources, cosmopolitanism, peer pressure, and external polices and incentives). Readiness for implementation with the self-designed IRT was only one construct of ‘inner setting’ we used to get an overview of implementation progress across the sites. Local implementation teams experienced the implementation programme as very successful in changing the culture of ICU professionals about delirium as indicator of brain failure and a problem that needs to be actively addressed, but that was not directly related to the degree of local implementation success.

One of the problems when comparing the degree of adherence with other guidelines implementation studies relates to the definitions of different PI measures^11^ and the measurement of total or partial compliance in relation to hospital survival.^13^ The question remains: when are we satisfied with the degree of adherence? We defined a target level for the PI for delirium screening only, and did not define this for other PIs or overall implementation success in advance. The definition of targeted adherence level in advance is not a common practice in implementation studies, but we suggest that this may provide more clarity on the goals of implementation, which may facilitate adherence and, ultimately, quality of care.^24^

Limitations of the study particularly relate to lacking assessment of the implementation context, for example, ICU culture and context of organisation in advance. Second, assessment of exposures of the ICUs to the implementation programme partly depended on self-reported assessments, which may not have been entirely accurate. Third, predefined knowledge level of >70% was a choice and may not have represented sufficient knowledge. A questionnaire was not validated and may not have had the optimal validity to test knowledge. Furthermore, ideally paired t-tests have been used for matching the responses before versus after implementation, but because we could not match the survey responses at the responder level, paired analysis was not possible and we reported only the crude numbers, omitting p values. Fourth, our design was not appropriate for measuring the association between the individual implementation strategies and adherence changes. Finally, experience with implementation was measured only among the local implementation team members. Also, the managers were not involved during the implementation whereas previous studies have shown that managers may play an important role in facilitating implementation.^25^ More inclusive assessment of experiences of healthcare professionals and managers with the implementation could have provided more information about the ‘why’ of non- (or suboptimal) adherence.

## Conclusions

Multifaceted implementation interventions can improve and sustain delirium guideline adherence in the ICU. Delivering multifaceted implementation interventions is feasible. Indicators of success or failure of the implementation remain very challenging to identify because of the multitude of factors influencing guideline adherence and clinical outcomes, including ICU culture which we did not formally assess. In spite of a general level of tailoring, variability in delirium guideline adherence at individual ICUs remained. For future quality improvement, this could possibly be resolved by investing in a higher degree of tailoring implementation interventions to ICUs’ local inner and outer contexts.
