# Supplementary material for: Prospective multicentre multifaceted before-after implementation study of ICU delirium guidelines: a process evaluation
Source: BMJ Open Qual. 2020 Sep 18;9(3):e000871. doi: 10.1136/bmjoq-2019-000871 (PMC7511605; doi:10.1136/bmjoq-2019-000871)
Supplement: Supplementary data [file bmjoq-2019-000871supp001.pdf]

Supplementary files for:

Prospective Multicenter Multifaceted Before-After Implementation Study of ICU Delirium Guidelines: a Process Evaluation

1. **Supplementary file 1:** Title: The completed StaRI checklist
2. **Supplementary file 2:** Title: Semi structured interview for the assessment of experiences of local implementation teams with the implementation
3. **Supplementary file 3**: Title: Patient Demographics and Baseline Clinical Characteristics in T4
4. **Supplementary file 4:** Title: Changes in Pain Agitation Delirium (PAD) Guidelines Performance Indicators at ICUs level across study
5. **Supplementary file 5:** Title: Clinical Outcomes at ICUs level across the study
6. **Supplementary file 6:** Title: Demographics of survey respondents
7. **Supplementary file 7:** Title: Experiences with the implementation program

Supplementary file 1: Title: The completed StaRI checklist

StaRI

Standards for Reporting Implementation Studies: the StaRI checklist for completion

The StaRI standard should be referenced as: Pinnock H, Barwick M, Carpenter C, Eldridge S, Grandes G, Griffiths CJ, Rycroft-Malone J, Meissner P, Murray E, Patel A, Sheikh A, Taylor SJC for the StaRI Group. Standards for Reporting Implementation Studies (StaRI) statement. *BMJ* 2017;356:i6795

The detailed Explanation and Elaboration document, which provides the rationale and exemplar text for all these items is: Pinnock H, Barwick M, Carpenter C, Eldridge S, Grandes G, Griffiths C, Rycroft-Malone J, Meissner P, Murray E, Patel A, Sheikh A, Taylor S, for the StaRI group. Standards for Reporting Implementation Studies (StaRI). Explanation and Elaboration document. *BMJ Open* 2017 2017;7:e013318

Notes: A key concept of the StaRI standards is the dual strands of describing, on the one hand, the implementation strategy and, on the other, the clinical, healthcare, or public health intervention that is being implemented. These strands are represented as two columns in the checklist.

The primary focus of implementation science is the implementation strategy (column 1) and the expectation is that this will always be completed.	The evidence about the impact of the intervention on the targeted population should always be considered (column 2) and either health outcomes reported or robust evidence cited to support a known beneficial effect of the intervention on the health of individuals or populations.

The StaRI standardsrefers to the broad range of study designs employed in implementation science. Authors should refer to other reporting standards for advice on reporting specific methodological features. Conversely, whilst all items are worthy of consideration, not all items will be applicable to, or feasible within every study.

Checklist item		Reported on page #	Implementation Strategy	Reported on page #	Intervention
			"Implementation strategy" refers to how the intervention was implemented		"Intervention" refers to the healthcare or public health intervention that is being implemented.
**Title and abstract**					
Title	1	1	Identification as an implementation study, and description of the methodology in the title and/or keywords		
Abstract	2	2	Identification as an implementation study, including a description of the implementation strategy to be tested, the evidence-based intervention being implemented, and defining the key implementation and health outcomes.		
**Introduction**					
Introduction	3	4	Description of the problem, challenge or deficiency in healthcare or public health that the intervention being implemented aims to address.		
Rationale	4	4	The scientific background and rationale for the implementation strategy (including any underpinning theory/framework/model, how it is expected to achieve its effects and any pilot work).	4	The scientific background and rationale for the intervention being implemented (including evidence about its effectiveness and how it is expected to achieve its effects).

**Results**					
Characteristics	17	8	Proportion recruited and characteristics of the recipient population for the implementation strategy	9	Proportion recruited and characteristics (if appropriate) of the recipient population for the intervention
Outcomes	18	8/9/10	Primary and other outcome(s) of the implementation strategy	9	Primary and other outcome(s) of the Intervention (if assessed)
Process outcomes	19	8/9/10	Process data related to the implementation strategy mapped to the mechanism by which the strategy is expected to work		
Economic evaluation	20	n.a.	Resource use, costs, economic outcomes and analysis for the implementation strategy	8	Resource use, costs, economic outcomes and analysis for the intervention
Sub-group analyses	21	9/10	Representativeness and outcomes of subgroups including those recruited to specific research tasks		
Fidelity/ adaptation	22	8/9	Fidelity to implementation strategy as planned and adaptation to suit context and preferences	8/9	Fidelity to delivering the core components of intervention (where measured)
Contextual changes	23	n.a.	Contextual changes (if any) which may have affected outcomes		
Harms	24	n.a.	All important harms or unintended effects in each group		
**Discussion**					
Structured discussion	25	11/12/13	Summary of findings, strengths and limitations, comparisons with other studies, conclusions and implications		
Implications	26	11/12/13	Discussion of policy, practice and/or research implications of the implementation strategy (specifically including scalability)	11/12	Discussion of policy, practice and/or research implications of the intervention (specifically including sustainability)
**General**					
Statements	27	3	Include statement(s) on regulatory approvals (including, as appropriate, ethical approval, confidential use of routine data, governance approval), trial/study registration (availability of protocol), funding and conflicts of interest		

Supplementary file 2: Title: Semi structured interview for the assessment of experiences of local implementation teams with the implementation

Supplementary file 2: Semi structured interview for the assessment of experiences of local implementation teams with the implementation

1. Do you think that the implementation of delirium directive (generally) was successful?
    - If not, why it was not successful?
2. Which components of the implementation were successful?
    - If yes, which:
    - If not, which:
3. Are the barriers identified at the beginning of the study for your center / ICU sufficiently resolved with the chosen implementation interventions?
4. Which individual components of the strategies have been effective and which ones (i.e. why the implementation was less successful (open question thus, and own opinion about this, will also provide additional information)?
5. Did you have a local project team / delirium expert team,
    - Who was involved?
    - How were the roles / responsibilities distribution inside the local team?
    - Had we had to tackle different things (study team and ICs) differently?
6. Describe Part 1: implementation of screening and
7. Describe Part 2: Implementation of prevention and treatment.
8. Is the guideline delirium sufficiently guaranteed, and what does this prove?
9. What are the thoughts about Feedback on delirium incidence and delirium screening?
10. Control for screening of delirium: Are you going through this and how?
11. Nursing - doctor cooperation?
12. Is the delirium App applicable in practice?

<u>Question about project organization:</u>

1. Were the objectives of the coordination team (study team / we) clearly / concretely formulated?
2. What do you think of time investment (e.g. to implement screening)?
3. Sufficient support from coordinating team to achieve goals?
4. What did this project teach you for future implementation projects (such as protocols, guidelines)?

- Organization,
- Material,
- Communication,
- Staff,
- time

What combinations of strategies have been essential to your practice (what has been the key to success?)
Process
Finally, complete the completed IRT table of the relevant hospital and complete it at the end the interview.
Also check for any structural changes to the IC have been made.

Supplementary file 3: Patient Demographics and Baseline Clinical Characteristics in T4

Characteristic	*Data-collection period[c] T4 / Sustaining*
**No. of patients, n**	519
**No. of ICU[a] days, n**	2727
**Gender, n (%)**	
Male	300 (58)
Female	219 (42)
**Age (years), median (IQR[a])**	66 (55, 76)
**Admission status, n (%)[b]**	
Elective surgery	135 (26)
Emergency surgery	55 (11)
Medical	271 (52)
**APACHE-II[a], median (IQR)**	16 (12, 22)
**Mechanically Ventilated patients, n (%)**	261 (51)
**Hospital, n (%)**	
1	73 (14)
2	117 (23)
3	103 (20)
4	37 (7)
5	124 (24)
6	65 (13)

[a]Acute Physiology and Chronic Health Evaluation II range is 0-71, IQR: Interquartile range; ICU: intensive care unit
[b] Admission status missing's for Sustaining period = 1
[c] Data about previous three phases were published previously[1]

1. Trogrlic Z, van der Jagt M, Lingsma H, Gommers D, Ponssen HH, Schoonderbeek JFJ, et al. Improved Guideline Adherence and Reduced Brain Dysfunction After a Multicenter Multifaceted Implementation of ICU Delirium Guidelines in 3,930 Patients. Crit Care Med. 2019.

Supplementary file 4: Changes in Pain Agitation Delirium (PAD) Guidelines Performance Indicators at ICUs level across study

Performance Indicator (PI)[1]	ICU	T1[2] baseline	T2	T3	T4 follow-up	Δ T1 - T4% (T4% - T1%)
**Delirium Screening** (Total No. of days with at least one CAM-ICU or ICDSC assessment recorded / Total No. of patient-days at ICU)	1	82	97	96	97	+15 (97 - 82)
	2	92	95	99	89	-3 (89 - 92)
	3	16	81	89	95	+79 (95 - 16)
	4	0	88	77	93	+93 (93 - 0)
	5	0	100	100	93	+93 (93 - 0)
	6	0	94	100	88	+88 (88 - 0)
	ALL	35	93	96	92	+57 (92 - 35)
**Sedation Assessments** (Total No. of days with at least one sedation assessment recorded / Total No. of ICU days in ventilated patients receiving sedation and /or opioids)	1	98	97	96	98	0 (98 - 98)
	2	93	96	99	90	-3 (90 - 93)
	3	61	88	78	46	-15 (46 - 61)
	4	51	99	78	94	+43 (94 - 51)
	5	99	100	100	100	+1 (100 - 99)
	6	85	75	70	65	-20 (65 - 85)
	ALL	86	94	90	86	0 (86 - 86)
**Light Sedation** (No. of light sedation days[3] / Total No. of ICU days in ventilated patients receiving sedation and /or opioids)	1	84	66	75	71	-13 (71 - 84)
	2	83	81	91	77	-6 (77 - 83)
	3	51	65	67	55	+4 (55 -51)
	4	25	65	49	71	+46 (71 - 25)
	5	63	70	72	72	+9 (72 - 63)
	6	37	30	33	43	+6 (43 - 37)
	ALL	55	58	61	62	+7 (55 - 62)
**Avoiding Benzodiazepines Sedation** (No. of benzodiazepines[4] sedation days / Total no. of ICU days in mechanically ventilated patients during at least one ICU-day AND having received sedation and/or opioids)	1	58	69	86	68	+10 (68 - 58)
	2	92	92	95	92	0 (92 - 92)
	3	56	60	83	86	+30 (86 - 56)
	4	96	98	93	83	-13 (83 - 96)
	5	37	39	55	52	+15 (52 - 37)
	6	13	23	95	97	+84 (97 - 13)
	ALL	64	69	83	82	+18 (82 - 64)
**No-Analgesia first sedation** (No. of patient without-analgesia-while-sedated days / Total number of patient sedation days)	1	48	45	39	22	-26 (22 - 48)
	2	6	12	14	15	+9 (15 - 6)
	3	19	17	20	45	+26 (45 - 19)
	4	9	23	12	11	+2 (11 - 9)
	5	27	14	19	23	-4 (23 - 27)
	6	11	16	9	15	+4 (15 - 11)

	ALL	22	21	20	19	-3 (19 – 22)
**Performing Physical Therapy** (No. of patient-days with PT / Total No. of patient ICU days; included with LOS > 2 days)	1	48	45	39	22	-26 (22-48)
	2	12	24	25	30	+18 (30 - 12)
	3	87	89	95	94	+7 (94 - 87)
	4	87	59	57	52	-35 (52 - 87)
	5	6	34	36	27	+21 (27 - 6)
	6	4	68	82	27	+23 (27 - 4)
	ALL	21	45	48	38	+17 (38 - 21)
**Performing Mobilization** (No. of patient-days with mobilization / Total No. of patient ICU days included with LOS > 2 days)	1	22	19	29	32	+10 (32 - 22)
	2	8	11	13	22	+14 (22 -8)
	3	26	30	33	45	+26 (45 - 26)
	4	10	18	16	20	+10 (20 - 10)
	5	4	4	5	2	-2 (2 - 4)
	6	6	16	30	20	+14 (20 - 6)
	ALL	10	14	19	23	+13 (23 - 10)

[1] Predefined Performance Indicator(s) were used to assess the Pain Agitation Delirium (PAD) guidelines recommendations. Weighted percentages of the total ICU patient days contributed by each ICU of all performance indicators for all four measurement periods are given.

[2] T1= Baseline measurement (Before the start of implementation); T2= After delirium screening implementation; T3= After PAD guidelines implementation; T4= follow-up 6 months after implementation.

[3] Definition of Light sedation: Richmond Agitation and Sedation Scale (RASS) >- 3 or Critically Ill Assessment Scale (CIA) >6 or Ramsay Sedation Scale <5, see manuscript text for references.

[4] Benzodiazepines = midazolam and / or lorazepam as continuous intravenous sedative.

Supplementary file 5: Clinical Outcomes at ICUs level across the study

Outcomes	Crude analysis												
		T1		T2			T3			T4			
	ICU	Patients, n		Patients, n		p-value (T1 versus T2)[a]	Patients, n		p-value (T1 versus T3)[a]	Patients (n)		p-value (T3 versus T4)[a]	p-value ALL[b]
Delirium duration (days), median (IQR)	1	59	3 (2, 6)	38	1.5 (1 - 5.3)	.03	44	3 (1 - 4)	0.11	24	4 (2 - 6.5)	0.19	0.08
	2	71	3 (2, 10)	83	2 (1 - 5)	.006	99	3 (1 - 6)	.036	36	4.5 (2 - 11)	.007	.002
	3	44	2 (1, 3)	60	2 (1 - 3)	0.35	54	2 (1 - 3)	0.49	23	2 (1 - 4)	0.63	0.78
	4	29	2 (1, 3)	30	2 (1 - 4)	0.68	15	2 (1 - 4)	0.68	7	2 (1 - 6)	0.82	0.85
	5	39	4 (2, 6)	38	1.5 (1 - 2)	<.001	59	1 (1 - 3)	<.001	27	1 (1 - 2)	0.53	<.001
	6	32	3 (1, 4.8)	51	1 (1 - 2)	.001	48	1 (1 - 2)	<.000	18	2 (1 - 3)	0.28	.001
	ALL	274	3 (2, 5)	300	2 (1 - 3)	<.001	319	2 (1 - 3)	<.001	135	2 (1 - 5)	0.92	<.001
Patients with delirium during ICU admission, n (%)	1	145	59 (41%)	151	38 (25%)	.004	188	44 (23%)	.001	72	24 (33%)	0.10	.003
	2	247	71 (29%)	242	83 (34%)	0.19	240	99 (41%)	.004	108	36 (33%)	0.16	.036
	3	231	44 (19%)	223	60 (27%)	.046	240	54 (23%)	0.36	102	23 (23%)	0.99	0.26
	4	158	29 (18%)	150	30 (20%)	0.71	73	15 (21%)	0.69	36	7 (19%)	0.89	0.98
	5	251	39 (16%)	271	38 (14%)	0.63	216	59 (27%)	.002	121	27 (22%)	0.31	.001
	6	305	32 (11%)	297	51 (17%)	.017	216	48 (22%)	<.001	62	18 (29%)	0.27	<.001
	ALL	1337	274 (21%)	1334	300 (21%)	0.21	1173	319 (27%)	<.001	501	135 (27%)	0.92	<.001
Duration of mechanical ventilation (days), median (IQR)	1	47	2 (1 – 3.6)	75	3 (2 – 6)	.005	84	3 (2 – 5)	.020	34	3 (2 – 9)	0.40	.023
	2	193	1 (1 – 3)	176	2 (1 – 5)	.006	192	2 (1 – 5)	<.001	96	3 (1.5 – 8)	.048	.003
	3	57	1 (1 – 3)	76	2 (1 – 3.5)	0.10	58	2 (1 – 3)	0.28	23	3 (2 – 5.5)	0.06	0.06
	4	50	1 (1 - 2.5)	38	2 (2 – 6)	.002	41	3 (2 – 8)	<.001	12	4 (1 – 5)	0.56	<.001
	5	120	2.6 (1 – 6.7)	103	3 (2 - 6)	0.12	118	3 (1 - 6)	0.62	59	3 (1.5 – 5)	0.72	0.42
	6	93	2.2 (1 – 6.4)	73	3 (2 – 7)	0.15	100	2 (1 - 4)	0.31	37	2 (2 – 4)	0.39	0.08
	ALL	560	1.5 (1 – 4.6)	541	2 (1 - 6)	<.001	593	2 (1 - 5)	<.001	261	3 (2 – 6)	.042	<.001
ICU LOS (days), median (IQR)	1	145	3 (2 – 6)	155	3 (2 - 5)	.033	195	3 (2 – 5)	.04	73	4 (2 – 6)	.01	.004
	2	247	3 (2 – 6)	248	3 (2 - 6)	0.94	242	3 (2 – 7)	0.65	117	3 (2 – 8)	0.45	0.98
	3	231	2 (2 - 3)	251	2 (2 – 4)	0.84	249	2 (2 – 4)	0.12	103	2 (2 – 3)	0.49	0.43
	4	158	2 (2 – 4)	166	2 (2 – 3)	.003	76	3.5 (3 – 6.5)	<.001	37	3 (2 – 5)	0.05	<.001
	5	251	3 (2 – 6)	271	3 (2 – 4)	.028	216	3 (2 – 5.5)	0.98	124	3 (2 - 4)	0.07	.038
	6	305	2 (2 – 4)	308	2 (2 – 3)	0.40	216	3 (2 – 4)	.020	65	4 (3 – 6)	<.001	<.001
	ALL	1337	3 (2 - 5)	1399	2(2 – 4)	<.001	1194	3 (2 - 5)	.003	519	3 (2 -5)	0.21	<.001
ICU Mortality, n (%)	1	145	14 (9.7%)	155	15 (9.7%)	0.99	195	18 (9.2%)	0.90	73	9 (12.3%)	0.45	0.90
	2	247	51 (20.6%)	248	42 (16.9%)	0.29	241	41 (17%)	0.31	117	14 (12%)	0.21	0.23
	3	231	9 (3.9%)	251	26 (10.4%)	.006	249	18 (7.2%)	0.11	103	8 (7.8%)	0.86	0.06
	4	158	10 (6.3%)	166	11 (6.6%)	0.91	75	8 (10.7%)	0.25	37	5 (13.5%)	0.66	0.34
	5	251	24 (9.6%)	271	23 (8.5%)	0.67	216	28 (13%)	0.24	124	21 (16.9%)	0.32	0.06
	6	305	27 (8.9%)	307	23 (7.5%)	0.54	216	13 (6.0%)	0.23	65	7 (10.8%)	0.19	0.52
	ALL	1337	135 (10.1%)	1398	140 (10%)	0.94	1192	126 (10.6%)	0.70	519	64 (12,3%)	0.29	0.49
Hospital Mortality, n (%)	1	145	32 (22.1%)	155	25 (16.1%)	0.19	195	27 (13.8%)	.048	69	9 (13%)	0.88	0.18
	2	247	59 (23.9%)	248	49 (19.8%)	0.27	242	55 (22.7%)	0.76	85	15 (17.6%)	0.33	0.53
	3	231	17 (7.4%)	251	45 (17.9%)	.001	249	30 (12%)	0.08	101	11 (10.9%)	0.76	.005
	4	158	21 (13.3%)	166	20 (12%)	0.74	76	14 (18.4%)	0.30	26	6 (23.1%)	0.61	0.32
	5	251	45 (17.9%)	271	50 (18.5%)	0.88	216	46 (21.3%)	0.36	120	33 (20.3%)	0.20	0.14
	6	305	42 (13.8%)	308	37 (12%)	0.51	216	22 (10.2%)	0.22	63	10 (15.9%)	0.21	0.53
	ALL	1337	216 (16.2)	1399	226 (16.2)	0.99	1194	194 (16.2)	0.95	464	84 (18.1%)	0.36	0.77

[a] P-values are calculated as difference between two independent groups with Mann-Whitney Test for continue outcomes and Pearson Chi-Square Test was used for bivariate outcomes.

[b] P-values are calculated as difference between four independent groups (marked as ALL) with Kruskal-Wallis Test for continue outcomes and Pearson Chi-Square Test was used for bivariate outcomes.

Supplementary file 6: Demographics of survey respondents

**Survey**	**BEFORE** n (%)	**AFTER** n (%)
Type of healthcare professional		
ICU-physicians	53 (14)	53 (20)
· Intensivists (including fellows)	37 (10)	38 (14)
· Residents	16 (4)	15 (16)
ICU Nurses	283 (79)	201 (76)
Delirium experts (psychiatrists, geriatricians and specialized psychiatric nurses)	24 (7)	10 (4)
Years of work experience [a]		
< 1	47 (13)	22 (8)
1 to 4	64 (18)	50 (19)
5 to 9	72 (20)	63 (24)
≥10	177 (49)	129 (49)
Working assignment [b]		
<35%	7 (2)	3 (1)
35-55%	28 (8)	19 (7)
55-75%	46 (13)	49 (19)
75-90%	93 (26)	76 (29)
90-100%	186 (52)	117 (44)
Age (years) [c]		
<25	16 (4)	2 (1)
25-34	109 (30)	87 (33)
35-44	87 (24)	63 (24)
45-54	99 (28)	72 (37)
>55	42 (12)	33 (13)
missing	6 (2)	7 (3)

Differences between 6 participating ICUs in first survey (before): [a] p=0.67, [b] p=0.79, [c] p=0.15
Differences between 6 participating ICUs in second survey (after): [a] p=0.26, [b] p=0.29, [c] p=0.0

Supplementary file 7: Experiences with the implementation program

Overall, the members of the local implementation teams experienced the implementation program as very successful. More in detail, this was mainly due to constant attention given to the different parts of the guideline by the implementation teams. The implementation management team was able to encourage local implementation teams to stay focused on implementation at their ICUs. Initially, attention from the implementation management team was sometimes perceived as intrusive, but this feeling waned over time. The feeling that delirium is a form of *"vital organ failure"* was an important message which was embraced by the ICU professionals. Gradually, delirium was seen as a problem that needs as much attention as other forms of organ failure in critically ill patients, such as renal failure, respiratory (lung) failure, etc. This was perceived as a '*change of culture*'. Two ICUs had tried to implement delirium screening in the past. However, the local team members stated that "*this round was much more successful,*" (than previous attempt and relating this mainly to the analysis of barriers for screening being done before the implementation program). Further, bedside-teaching (practical training of delirium screening), creating a firm basis for acceptance and support, optimizing ICT facilities for screening and treatment, developing a comprehensive protocol and acceptance into daily rounds of the ICU were regarded facilitators for the implementation in some centers that succeeded in these items. The lack of ICT facilities and Research Nurses turnover were regarded crucial factors that limited the implementation at ICU 4. The respondents indicated that the implementation process sometimes faltered in their organization. For these local implementation leaders, the Implementation Readiness Test (IRT) was a very useful tool and worked for them as an "*implementation thermometer*" to accelerate the process. In addition, although the implementation took considerable time investment from the local teams, it had obviously translated into a concrete change of practice. At times, it was felt the local teams could have been addressed more actively by the implementation management team, referring to more directive and clearer clues on what to do and when. On the other hand, the project in different ICUs also had spin-off effects like optimizing collaboration with other disciplines. The implementation of other guideline recommendations can be picked up in the future because of the experience with this implementation (e.g. use of champions, opinion leaders (formally appointed an intensivist and research nurse at each site) and the use of IRT. Most people interviewed believed that delirium screening and drug treatment had been guaranteed in their ICU but that non-pharmacological interventions (such as earplugs) and other preventive measures still required attention for the future.
